# Top-Down Influences of the Medial Olivocochlear Efferent System in Speech Perception in Noise

**DOI:** 10.1371/journal.pone.0085756

**Published:** 2014-01-20

**Authors:** Srikanta K. Mishra, Mark E. Lutman

**Affiliations:** 1 Institute of Sound and Vibration Research, University of Southampton, Southampton, United Kingdom; 2 Department of Communication Sciences and Disorders, Butler University, Indianapolis, Indiana, United States of America; Utrecht University, Netherlands

## Abstract

One of the putative functions of the medial olivocochlear (MOC) system is to enhance signal detection in noise. The objective of this study was to elucidate the role of the MOC system in speech perception in noise. In normal-hearing human listeners, we examined (1) the association between magnitude of MOC inhibition and speech-in-noise performance, and (2) the association between MOC inhibition and the amount of contralateral acoustic stimulation (CAS)-induced shift in speech-in-noise acuity. MOC reflex measurements in this study considered critical measurement issues overlooked in past work by: recording relatively low-level, linear click-evoked otoacoustic emissions (CEOAEs), adopting 6 dB signal-to-noise ratio (SNR) criteria, and computing normalized CEOAE differences. We found normalized index to be a stable measure of MOC inhibition (mean = 17.21%). MOC inhibition was not related to speech-in-noise performance measured without CAS. However, CAS in a speech-in-noise task caused an SNR_SP_ enhancement (mean = 2.45 dB), and this improvement in speech-in-noise acuity was directly related to their MOC reflex assayed by CEOAEs. Individuals do not necessarily use the available MOC-unmasking characteristic while listening to speech in noise, or do not utilize unmasking to the extent that can be shown by artificial MOC activation. It may be the case that the MOC is not actually used under natural listening conditions and the higher auditory centers recruit MOC-mediated mechanisms only in specific listening conditions–those conditions remain to be investigated.

## Introduction

The “top-down” mechanisms in the auditory system involve modulation of the auditory periphery by higher centers in the brain via the efferent pathway [1]. With the discovery of otoacoustic emissions (OAEs) [2], the medial olivocochlear (MOC) efferents have received special attention [3], [4]. The MOC fibers project from the superior olivary complex to innervate cochlear outer hair cells (OHCs) via cholinergic synapses. Physiologically, MOC system activation causes OHC hyper-polarization, thus inhibiting OHC electromotility and reducing gain of the cochlear amplifier [3]. This, in turn, alters OAE amplitude and phase characteristics and is called MOC reflex or inhibition [3], [4]. We use the terms MOC inhibition and reflex interchangeably in this report. One of the putative roles of the MOC efferents is optimizing the perception of signals in background noise. Animal work suggests a “MOC unmasking” hypothesis in which MOC system activation reduces cochlear responses to continuous noise, allowing greater responsiveness to transient acoustic signals embedded in the noise [5]–[7].

In humans, while the MOC activation is found to be linked with performance on “simpler” psychoacoustic tasks, such as tone detection and intensity discrimination in noise [8]–[13], its relationship with speech perception in noise remains equivocal. Some studies reported a positive correlation between MOC inhibition and speech recognition in noise [11], [14]–[18], one study reported the opposite effect [19] and others have failed to establish this association [20]–[23]. The interpretation of these results is complicated by potential confounding variables related to MOC inhibition measurements. These studies have either used click-evoked (CE) or distortion product (DP) OAEs with contralateral acoustic stimulation (CAS) to measure the MOC reflex. Several factors that are currently known need to be considered for precise measurement of the MOC reflex [3], [4], e.g., (1) non-linear click method for recording click-evoked OAEs (CEOAEs), (2) signal-to-noise ratio (SNR) of the OAE responses, (3) distortion product OAEs (DPOAEs) fine structure and component mixing, (4) raw or not normalizing OAE differences, (5) low-level middle-ear muscle reflexes (MEMRs), and (6) level and bandwidth of the broadband noise (BBN) elicitor.

The aforementioned studies that attempt to probe the role of MOC efferents in speech perception in noise have largely overlooked critical methodological issues. Most CEOAE-based studies applied a liberal 3 dB SNR criterion, while others did not report the SNR [14], [15], [17]–[20]. Some reports either did not measure MEMRs [14], [15], [17], [20], [23] or used high click levels to record OAEs that may have evoked MEMRs [15], [17]. A few studies employed the conventional non-linear stimulus paradigm to record CEOAEs [15], [17]; this method cancels out most of the reflection emission, possibly limiting the MOC reflex magnitude. Numerous studies did not report the bandwidth of the BBN elicitor used for MOC activation [15]–[18], [20], [22], [23]. Normalization of OAE differences was not attempted in any of these studies. Several DPOAE-based studies did not appropriately consider fine structure or component separation that is shown to influence the MOC reflex magnitude [16], [21], [22]. One study that accounted for fine structure controversially computed MOC reflex at the fine structure minima or dips [21]; these dips reflect spurious component mixing and are unrelated to MOC reflex strength [4], [24], [25].

In this report, we attempt to control crucial methodological confounds and revisit the “MOC unmasking” hypothesis by re-examining the relationship between MOC inhibition and speech perception in noise in normal hearing (NH) human listeners. Specifically, we tested two working hypotheses in the same NH individuals: (1) MOC inhibition should be associated with speech-in-noise performance and (2) MOC reflex magnitude is directly related with the amount of CAS-induced change in speech-in-noise acuity. MOC reflex testing in this study capitalized on significant advancements in this area [3], [4] by: recording the CEOAEs at a relatively low level, adopting a higher SNR (6 dB) criterion, and computing normalized OAE differences. Additionally, measures similar to a previous study [26] were taken to rule out the contamination of MOC reflex magnitude by MEMRs. In order to have higher face validity, speech-in-noise performance was measured with speech-shaped noise that better represents real-life listening situations.

## Methods

### Ethics Statement

The Institute of Sound and Vibration Research Human Experimentation Safety and Ethics Committee, University of Southampton approved the study protocol. Subjects were students at the University of Southampton. Written and informed consent was obtained from every subject.

### Subjects

Eighteen human adults (eight females and 10 males) between the ages of 21 and 30 years with normal hearing thresholds (15 dB HL or better at octave frequencies between 250–8000 Hz) and normal tympanograms (static acoustic admittance between 0.35 and 1.75 mmho and peak pressure between +50 to −100 daPa) in both ears participated in this study. Experiments were conducted on one ear per subject (right ears only). All subjects included in this study had ipsilateral acoustic reflex thresholds (ARTs) greater than 60 dB SPL for BBN elicitors and had CEOAEs with at least 6 dB SNR (actual SNRs ranged from 8 to 12 dB; procedures detailed below). Physiologic and perceptual measurements were conducted in all subjects; the order of these tests was randomly selected and was counter-balanced between subjects. All subjects were native speakers of British English. Experimental sessions lasted from 90–130 minutes. All procedures were conducted inside an acoustically treated double-room setup.

The sample size (n = 18) was calculated by assuming a moderate correlation coefficient (*r = *0.56) and a power of 0.80 to find a statistically significant correlation between MOC inhibition and speech recognition performance at a 95% significance level (α = 0.05). The effect size was estimated from previous studies that reported a statistically significant correlation [14], [15], [18], [19].

### CEOAE-based Assay of MOC Inhibition

CEOAE instrumentation and procedures replicated those previously described in a published work [26]. CEOAEs were measured using the ILO 292 Echoport system with UGS transient-evoked OAE Probe (Otodynamics Ltd., London, UK). CEOAEs were recorded in a linear mode with 80 µs clicks presented at 57 dB pSPL (±0.3 dB; calibrated in an IEC-711 ear simulator using the peak-equivalent method with a 1 kHz reference tone) at a rate of 50/s. Recordings were time-windowed from 2 to 20 ms. Responses to a total of 260 sets of clicks were averaged above the noise rejection level of 47 dB. The ILO292 averages into two alternate buffers: A and B. Signal is estimated from the 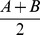 waveform and noise is estimated from the A–B difference waveform. Reproducibility is defined as the zero-lag correlation coefficient between A and B buffers. The ILO V6 Clinical OAE software records the stimulus level in the ear canal at the onset of testing and subsequently monitors the level throughout the response acquisition period to display stimulus stability. Responses were accepted only if the overall waveform reproducibility and stimulus stability exceeded 90% and 85% respectively.

Two sets of CEOAEs were recorded in 18 awake subjects: one without CAS and another with CAS. To assess immediate intra-subject repeatability of the MOC reflex indices, these two sets of recordings were repeated with probe tip reinsertion in the same test session in 13 subjects. The presentation order of CAS condition was randomized and was counter-balanced between subjects. The overall response amplitude of CEOAEs in dB SPL in each condition was measured directly from the ILO system that corresponds to the powers in the whole frequency region of the power spectrum for the time of the analysis.

Contralateral BBN (0.125–12 kHz) with a flat spectrum, inside the test cavity, was generated by a GSI 61 audiometer (Grason-Stadler Inc., Eden Prairie, Minnesota) and presented at 30 dB SL (re; BBN threshold) through an Etymotic ER-3A insert earphone. In the CAS condition, BBN was manually switched on prior to the onset of CEOAE recordings. CEOAE recordings were performed in a passive listening condition and subject instructions were similar to a previous report [26].

The MOC inhibition was quantified by two indices: (1) raw dB effect (ΔCEOAE) and (2) normalized index (ΔCEOAEn). For ΔCEOAE, the CEOAE amplitude in the CAS condition was subtracted from the baseline amplitude (without CAS). Positive values denote MOC inhibitory effects. Past work has typically used this index. We do not use this index to interpret the data or discuss the results because it may introduce biases (see Figures S1 and S2 in Supporting Information). The ΔCEOAEn is the change (in linear scale) in CEOAE amplitude due to CAS normalized to baseline amplitude and was quantified as a percentage change from the baseline amplitude [26]. Referencing to baseline amplitude eliminates biases related to inter-subject differences in magnitude of the CEOAE.

In order to rule out the possibility of MEMRs evoked by the contralateral BBN used for the MOC test, ARTs were measured for each subject using a GSI-TympStar (Grason-Stadler Inc., Eden Prairie, MN) following a previously reported procedure [26]. The threshold of audibility for BBN ranged from 18 to 27 dB SPL (mean = 24.8 dB SPL), and the ARTs ranged from 68 to 80 dB SPL (mean = 78.4 dB SPL). Because clinical instruments produce higher ARTs, a constant of 12 dB gleaned from wideband acoustic reflectance studies [27], [28] was applied to detect subclinical ARTs. The mean corrected ART was 66.4 dB SPL. Thus the BBN level used for the MOC reflex (mean = 54.8 dB SPL) was lower than the ARTs by a mean of 11.6 dB (or uncorrected 23.6 dB). This suggests that MEMRs had no or minimal influence on the present MOC inhibition measurements.

### Speech Recognition in Noise

Speech recognition in noise was measured using a computerized version of the Four Alternative Auditory Feature (FAAF) test [29]. This is a forced-choice word recognition task consisting of 20 sets of four binary and minimally paired words (e.g., get, wet, bet, yet), giving an 80-item list. The target word occurs in the context of the carrier phrase, “Can you hear (target) clearly?” The subject's task is to select the target word from the choice of four displayed in a touch-screen.

The FAAF materials were presented from a PC with a 24-bit sound card (Sound Blaster Audigy 4 Pro, Creative Labs, Inc., Milpitas, CA), at a sample rate of 44 kHz, routed via a Kamplex diagnostic audiometer to a TDH 50P earphone (Kamplex KC40, Interacoustics A/S, Denmark). They were presented against a background of steady noise that had been filtered to give a similar long-term spectrum to the target words and delivered by the same earphone. The speech presentation level was fixed at 60 dB SPL while the ipsilateral noise level was initially presented at 56 dB SPL and then varied adaptively in 2-dB steps. Speech recognition threshold in noise was determined using an adaptive technique to converge on an SNR targeting a 70.7% correct score by means of a two-up and one-down algorithm [30]. For brevity and to distinguish from SNR in OAEs, SNR in the speech-in-noise task is denoted as SNR_SP_. The SNR_SP_ is defined as the dB difference between word SPL and noise SPL. The 70.7% score was estimated from the mean of the final eight reversals in the adaptive procedure. Speech recognition threshold (SRT) in noise was measured in each subject’s right ear in two CAS conditions: (1) without CAS and (2) with CAS. The CAS-induced change in SRT (ΔSRT) was computed by subtracting SRT with CAS from that without CAS. The CAS procedures were similar to those in CEOAE measurements with an inclusion of 5–10 minute breaks between two conditions. SRT measurements were repeated in the same test session (n = 13) to assess immediate intra-subject repeatability of ΔSRT.

### Statistical Analysis

The Shapiro-Wilk tests determined that the data distribution could be Gaussian (ΔCEOAEn *W* statistic = 0.93, *p* = 0.28; SRT *W* statistic = 0.95, *p* = 0.42; ΔSRT *W* statistic = 0.95, *p* = 0.43). Immediate intra-subject repeatability was computed by dividing the standard deviation of the differences between two repeated measurements by √2. The reason for dividing by √2 is because the standard deviation of the differences includes the pooled uncertainty of the two measurements, and if each replication has the same uncertainty (intra-subject variance) the difference has double the variance. Intra-subject SD on replication confirmed repeatability of MOC reflex and ΔSRT indices (n = 13). Data from two measurements were averaged to enhance stability of the measures. Effect of CAS on SRT was assessed using a paired samples *t*-test. Pearson’s product moment correlation coefficients were computed to examine the relationship between (1) ΔCEOAEn and SRT and (2) ΔCEOAEn and ΔSRT. The alpha level was Bonferroni adjusted (0.025 = 0.05/2).

## Results

### Physiological Results

**Figure 1:**
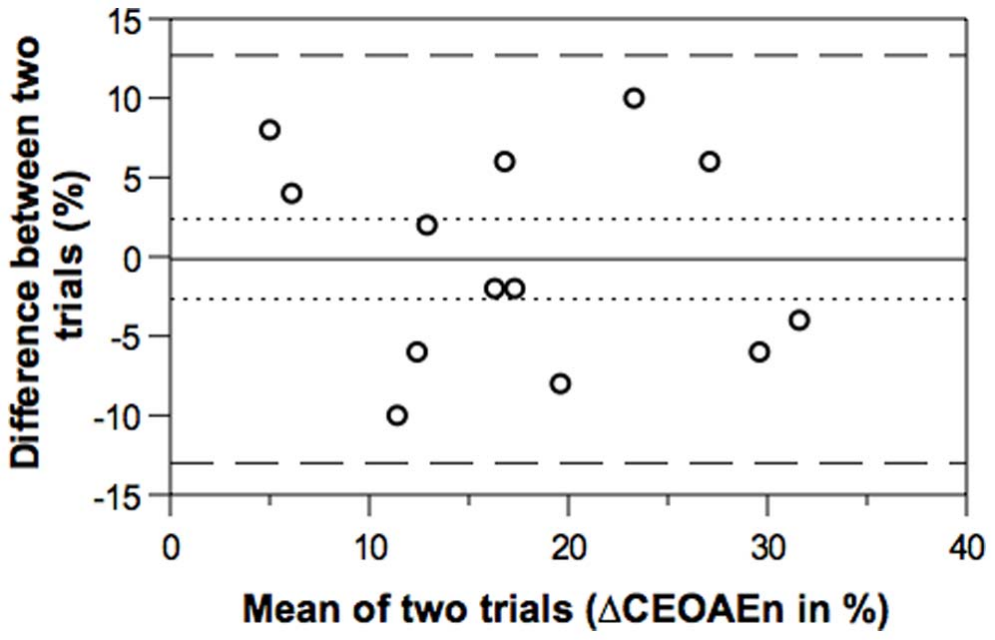
Bland and Altman plot for normalized index (ΔCEOAEn). Bland-Altman plot showing bias (solid line), 95% limits of agreement (heavy-dashed lines), and 95% confidence intervals (CIs) of bias (light-dashed lines). CEOAE indicates click-evoked otoacoustic emission; circles indicate repeated data (n = 13). The bias line shows average discrepancy between trials; the zero lying within 95% CIs of bias suggests no significant systematic error between trials; the limits of agreement represent the range of values in which agreement between trials may lie for approximately 95% of the sample. Note that intra-subject SD on replication for ΔCEOAE was 4.64%.

CEOAE recordings with and without CAS met the acceptable criteria (detailed in methods) in all subjects. Intra-subject SD on replication for ΔCEOAE and ΔCEOAEn were 0.35 dB and 4.64% (n = 13), respectively, implying good repeatability of our measurement procedures regardless of OAE probe re-fitting. The distribution of differences between the two sets of ΔCEOAEn measurements was computed using the Bland and Altman method [31], [32] and is shown in Figure 1. The mean raw dB effect and normalized index were 1.61 dB (SD = 0.92) and 17.21% (SD = 8.60), respectively. Overall, our MOC inhibition data are free from noise floor effects, followed a Gaussian distribution, and are repeatable.

### Perceptual Results

The mean SRT without CAS was –3.99 dB (SD = 1.75; n = 18), meaning that, on average, subjects could achieve 70.7% score when the speech signal was presented 3.99 dB below the noise level. A paired *t*-test revealed a statistically significant difference (t = 9.03; p<0.001) in SRT between two CAS conditions (with and without CAS) with an effect size of 2.01 [33]. The SRT lowered (or improved) with CAS (mean = –6.44 dB, SD = 1.79). The mean CAS-induced change in SRT was 2.45 dB (SD = 1.14; n = 18) with an intra-subject SD on replication of 0.61 dB (n = 13). This shows that with CAS subjects needed a less favorable SNR_SP_ to achieve the target score (70.7%).

### Correlation between Physiological and Perceptual Indices

**Figure 2:**
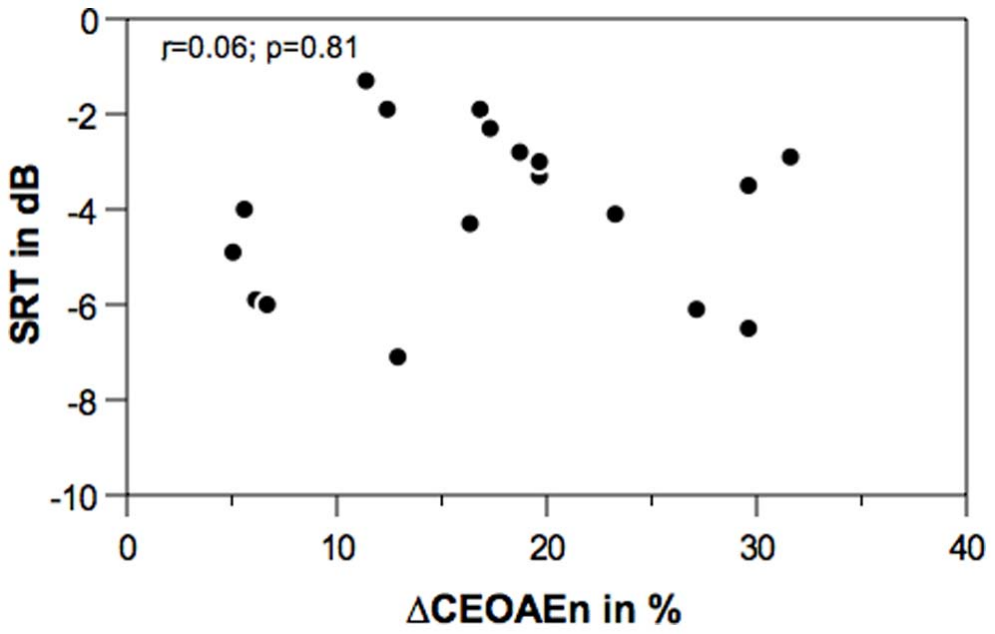
MOC inhibition and speech perception in noise. Bivariate scatterplot depicting the relationship between MOC reflex magnitude (ΔCEOAEn) and speech recognition threshold without CAS (SRT). Pearson’s correlation coefficient (r) is inserted on top left corner of the plot.

**Figure 3:**
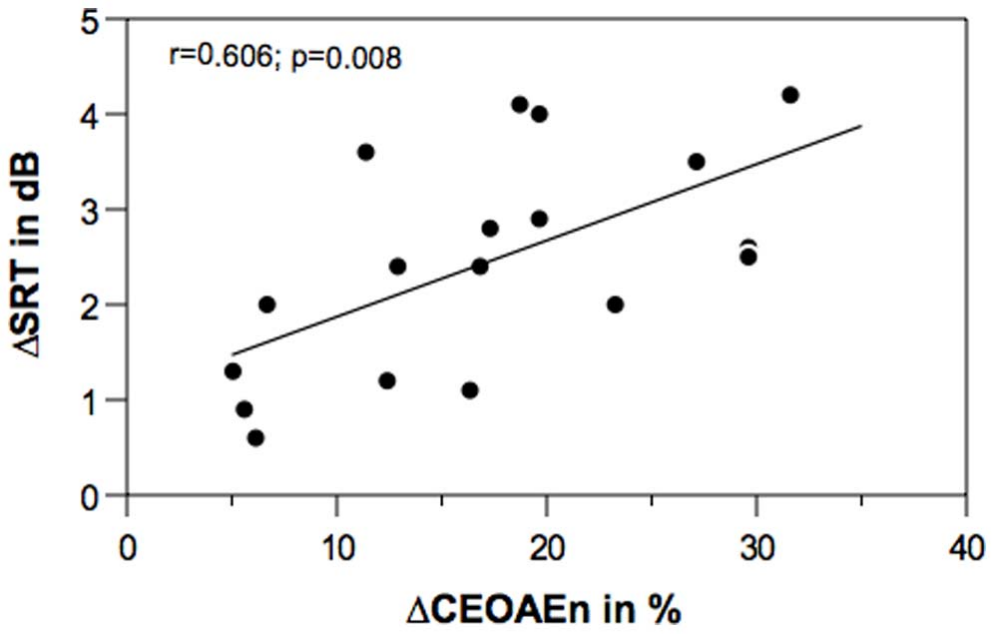
MOC inhibition and CAS-induced SNR_SP_ enhancement. The CAS-induced shift in SRT is plotted as a function of MOC reflex magnitude (ΔCEOAEn). A linear regression line is inserted to show the predictive relationship. Pearson’s correlation coefficient (r) is inserted on top left corner of the plot.

Speech recognition threshold without CAS and CAS-induced change in SRT as a function of normalized MOC index are shown in bivariate scatterplots, Figures 2 and 3, respectively. Pearson’s product moment correlation analysis showed no significant correlation between ΔCEOAEn and SRT without CAS (*r* = 0.06; n = 18; *p* = 0.81). Contrary to our hypothesis, ΔCEOAEn did not correlate with speech recognition in noise. There was a statistically significant correlation between ΔCEOAEn and ΔSRT (*r* = 0.606; n = 18; *p* = 0.008). An inspection of Figure 3 (with regression line) indicates that the calculated significant correlation coefficient reflects contributions of all data points. Collectively, correlation analyses suggest that individuals with larger MOC inhibition (1) do not show better SRT or higher speech recognition in noise performance without CAS but nonetheless (2) exhibit larger CAS-induced change in SRT.

## Discussion

The present study was designed to reconcile the role of the MOC system in speech-in-noise perception after carefully considering critical MOC measurement issues discussed elsewhere [3], [4], [26]. The reported MOC inhibition data are free from noise floor and MEMR effects, and can be modelled by a Gaussian distribution. Physiological and perceptual measurements showed high stability. These measurement caveats are often ignored in past work investigating the functional role of MOC; e.g., 15–17,20. The principal findings are: (1) the normalized MOC inhibitory effect (mean = 17.21%) is repeatable; (2) the magnitude of MOC inhibition, assayed by CEOAEs, is not related to speech-in-noise performance without CAS; (3) contralateral acoustic stimulation during speech-in-noise measurements caused a significant decrease in SRT (or enhancement in SNR_SP_); (4) MOC reflex magnitude positively correlated with CAS-induced change in speech-in-noise acuity. These novel results from the same listeners provide a unique opportunity for re-interpreting the top-down influences of the medial efferents in speech-in-noise perception. We approach the discussion in two realms: whether listeners use MOC-mediated mechanisms for understanding speech in noise and to what extent MOC inhibitory effects are capable of influencing speech-in-noise acuity.

### MOC-mediated Mechanisms in Speech-in-noise Perception

Contrary to our expectation, present results do not lend credence to the simple hypothesis that MOC inhibition improves overall speech-in-noise recognition–this hypothesis is convenient, but oversimplified and potentially problematic. Speech perception in noise is a complex process involving several auditory and non-auditory mechanisms (their discussion is beyond the present scope). The involvement and benefit of MOC-mediated (or MOC-unmasking) mechanisms in speech-in-noise perception appear to be task-dependent or stimuli/noise-dependent. For example, the MOC reflex correlated positively with /*bi*/−/*di*/ and negatively with /*da*/−/*ga*/ discrimination in noise [18], [19]. Compelling evidence suggests task-dependent attentional control of MOC inhibition [34], [35]. Present data suggest that the MOC effects on the cochlear encoding of click responses used to measure CEOAEs may be unrelated to whatever natural MOC effects that may modulate cochlear encoding of various speech tokens in noise. It is plausible that individuals may not essentially employ MOC-mediated mechanisms while listening in noise, hence, no observed relationship between MOC reflex magnitude and speech-in-noise performance. An alternative interpretation is that the tested perceptual condition (without CAS) elicited less-than-optimal or minuscule MOC activity, which was subdued by stronger non-MOC-mediated and non-auditory mechanisms; e.g., [36]. Recall that in the speech perception test (without CAS) the speech level was fixed at 60 dB SPL and noise type was speech-shaped; these acoustic signals may not be potent elicitors for the crossed MOC fibers [3], [4], and there may be little centrally-mediated MOC activity. In contrast, CAS optimally stimulated uncrossed fibers in perceptual measurement conditions where CAS was used.

### CAS-induced SNR_SP_ Enhancement

Contralateral acoustic stimulation with BBN caused a decrease in SRT. Individuals needed less favorable SNR_SP_ to achieve the target speech-in-noise recognition score. On average, this MOC-unmasking SNR_SP_ advantage was 2.45 dB, and corresponded to 12–16% improvement in speech recognition scores at typical conversation levels [29]. These results are consistent with emerging modelling efforts [37]–[39]. The present CAS-induced SNR_SP_ improvement is not due to release from masking that occurs when correlated noises are used binaurally [40]. Although interaural level differences can shift the perceived intracranial position of the image evoked by binaural uncorrelated noise [41], such an influence is not known to produce SNR_SP_ effects equivalent in size and distribution to that observed here–that could yield a correlation with MOC inhibition. A larger concern is the inability to eliminate potential binaural stimulation of the MOC efferents during speech recognition measurements with CAS. Note that tested listeners show normal MOC inhibitory characteristics per previous data [26].

Physiological results of MOC inhibition correlated with CAS-induced SNR_SP_ enhancements. As expected, individuals with stronger MOC inhibition showed larger CAS-induced improvement in SNR_SP_. Note that we used a normalized metric of MOC inhibition to test this relationship. Two perplexing findings here call for prudent interpretation. First, the MOC inhibition is not related with speech recognition in noise performance, and second, the MOC inhibition is positively related with CAS-induced SNR_SP_ improvement in the same listeners. An opportune construal is that MOC inhibition is capable of producing an SNR_SP_ enhancement; however, the auditory system may not use this resource in a reflexive manner, perhaps because its involvement may not be always beneficial [13], [19]. Rather, higher auditory structures in the brain may recruit MOC-mediated mechanisms only in specific listening situations, which remain largely elusive.

### Experimental Caveats

The basic premise of this and other studies investigating the role of MOC in speech perception is often oversimplified–seeking simple correlations between MOC inhibitory effect and measures of speech recognition in noise. In addition, our physiological measurements of MOC inhibition were conducted in a passive listening condition, whereas speech-in-noise perception testing reflects an active listening situation. The listening condition may influence the correlation between MOC inhibition magnitude and speech-in-noise recognition performance. However, the active versus passive condition difference did not preclude a correlation between MOC reflex magnitude and CAS-induced change in speech-in-noise acuity. Importantly, our CEOAE measurements may represent fast MOC effects [42], [43]. In real-life listening situations, a combination of both fast and slow effects may be possible.

## Conclusions

1. We found normalized index to be a stable measure of MOC inhibition. The mean magnitude of normalized MOC reflex was approximately 17% in normal-hearing adults.
2. Controlled MOC stimulation leads to an improvement in speech recognition in noise. On average, this advantage corresponded to an improvement of 2.45 dB SNR_SP_ at the stimulus/noise levels tested here. The magnitude of MOC inhibition of CEOAEs is positively correlated with CAS-induced SNR_SP_ enhancement in a speech-in-noise task.
3. Individuals do not necessarily use the available MOC-unmasking characteristic while listening to speech in noise, or do not utilize MOC unmasking to the extent that can be shown by artificial MOC activation. The listening conditions under which listeners utilize the MOC system without artificial activation to improve speech recognition in noise remain to be explored.

## Supporting Information

Figure S1 **MOC inhibition in raw dB index and speech perception in noise.** Bivariate scatterplot depicting the relationship between MOC reflex magnitude (ΔCEOAE) and speech recognition threshold without CAS (SRT). Pearson’s correlation coefficient (r) is inserted on top left corner of the plot.
(TIFF)

Figure S2 **MOC inhibition in raw dB index and CAS-induced SNR_SP_ enhancement.** The CAS-induced shift in SRT is plotted as a function of MOC reflex magnitude (ΔCEOAE). Pearson’s correlation coefficient (r) is inserted on top left corner of the plot.
(TIFF)

## References

[1] Xiao Z, Suga N (2002) Modulation of cochlear hair cells by the auditory cortex in the mustached bat. Nature Neuroscience 5: 57–63. 11753417 10.1038/nn786

[2] Kemp DT (1978) Stimulated acoustic emissions from within the human auditory system. Journal of the Acoustical Society of America 64: 1386–1391. 744838 10.1121/1.382104

[3] Guinan JJ (2006) Olivocochlear efferents: anatomy, physiology, function, and the measurement of efferent effects in humans. Ear and Hearing 27: 589–607. 17086072 10.1097/01.aud.0000240507.83072.e7

[4] Guinan JJ (2010) Cochlear efferent innervation and function. Current Opinion in Otolaryngology & Head and Neck Surgery 18: 447–453. 20717032 10.1097/MOO.0b013e32833e05d6 PMC3075443

[5] Winslow RL, Sachs MB (1988) Single-tone intensity discrimination based on auditory-nerve rate responses in backgrounds of quiet, noise, and with stimulation of the crossed olivocochlear bundle. Hearing Research 35: 165–189. 3198509 10.1016/0378-5955(88)90116-5

[6] Kawase T, Liberman MC (1993) Antimasking effects of the olivocochlear reflex. I. Enhancement of compound action potentials to masked tones. Journal of Neurophysiology 70: 2519–2532. 8120596 10.1152/jn.1993.70.6.2519

[7] Kawase T, Delgutte B, Liberman MC (1993) Antimasking effects of the olivocochlear reflex. II. Enhancement of auditory-nerve response to masked tones. Journal of Neurophysiology 70: 2533–2549. 8120597 10.1152/jn.1993.70.6.2533

[8] Micheyl C, Collet L (1996) Involvement of the olivocochlear bundle in the detection of tones in noise. Journal of the Acoustical Society of America 99: 1604–1610. 8819856 10.1121/1.414734

[9] Micheyl C, Morlet T, Giraud AL, Collet L, Morgon A (1995) Contralateral suppression of evoked otoacoustic emissions and detection of a multi-tone complex in noise. Acta Otolaryngologica 115: 178–182. 10.3109/00016489509139286 7610799

[10] Micheyl C, Perrot X, Collet L (1997) Relationship between auditory intensity discrimination in noise and olivocochlear efferent system activity in humans. Behavioral Neuroscience 111: 801–807. 9267657 10.1037//0735-7044.111.4.801

[11] Zeng FG, Martino KM, Linthicum FH, Soli SD (2000) Auditory perception in vestibular neurectomy subjects. Hearing Research 142: 102–112. 10748333 10.1016/s0378-5955(00)00011-3

[12] Bhagat SP, Carter PH (2010) Efferent-induced change in human cochlear compression and its influence on masking of tones. Neuroscience Letters 485: 94–97. 20813158 10.1016/j.neulet.2010.08.069

[13] Garinis A, Werner L, Abdala C (2011) The relationship between MOC reflex and masked threshold. Hearing Research 282: 128–137. 21878379 10.1016/j.heares.2011.08.007 PMC3242450

[14] Giraud AL, Garnier S, Micheyl C, Lina G, Chays A, et al (1997) Auditory efferents involved in speech-in-noise intelligibility. Neuroreport 8: 1779–1783. 9189932 10.1097/00001756-199705060-00042

[15] Kumar UA, Vanaja CS (2004) Functioning of Olivocochlear Bundle and Speech Perception in Noise. Ear and Hearing 25: 142–146. 15064659 10.1097/01.aud.0000120363.56591.e6

[16] Kim S, Frisina RD, Frisina DR (2006) Effects of age on speech understanding in normal hearing listeners: Relationship between the auditory efferent system and speech intelligibility in noise. Speech Communication 48: 855–862.

[17] Yilmaz ST, Sennaroğlu G, Sennaroğlu L, Köse SK (2007) Effect of age on speech recognition in noise and on contralateral transient evoked otoacoustic emission suppression. The Journal of Laryngology and Otology 121: 1029–1034. 17381896 10.1017/S0022215107006883

[18] De Boer J, Thornton ARD (2008) Neural correlates of perceptual learning in the auditory brainstem: efferent activity predicts and reflects improvement at a speech-in-noise discrimination task. The Journal of Neuroscience 28: 4929–4937. 18463246 10.1523/JNEUROSCI.0902-08.2008 PMC6670751

[19] De Boer J, Thornton ARD, Krumbholz K (2012) What is the role of the medial olivocochlear system in speech-in-noise processing? Journal of Neurophysiology 107: 1301–1312. 22157117 10.1152/jn.00222.2011 PMC3311680

[20] Harkrider AW, Smith SB (2005) Acceptable noise level, phoneme recognition in noise, and measures of auditory efferent activity. Journal of the American Academy of Audiology 16: 530–545. 16295240 10.3766/jaaa.16.8.2

[21] Wagner W, Frey K, Heppelmann G, Plontke SK, Zenner H-P (2008) Speech-in-noise intelligibility does not correlate with efferent olivocochlear reflex in humans with normal hearing. Acta Oto-laryngologica 128: 53–60. 17851961 10.1080/00016480701361954

[22] Mukari SZ-MS, Mamat WHW (2008) Medial olivocochlear functioning and speech perception in noise in older adults. Audiology Neurootology 13: 328–334. 10.1159/000128978 18460868

[23] Stuart A, Butler AK (2012) Contralateral suppression of transient otoacoustic emissions and sentence recognition in noise in young adults. Journal of the American Academy of Audiology 23: 686–696. 23072961 10.3766/jaaa.23.9.3

[24] Abdala C, Mishra SK, Williams TL (2009) Considering distortion product otoacoustic emission fine structure in measurements of the medial olivocochlear reflex. Journal of the Acoustical Society of America 125: 1584–1594. 19275316 10.1121/1.3068442 PMC2736726

[25] Deeter R, Abel R, Calandruccio L, Dhar S (2008) Changes in distortion product otoacoustic emission (DPOAE) fine structure due to contralateral acoustic stimulation. Journal of the Acoustical Society of America 126: 2413–2424. 10.1121/1.3224716 PMC2787069 19894823

[26] Mishra SK, Lutman ME (2013) Repeatability of click-evoked otoacoustic emission-based medial olivocochlear efferent assay. Ear and Hearing 34: 789–798. 23739244 10.1097/AUD.0b013e3182944c04

[27] Feeney MP, Keefe DH, Marryott LP (2003) Contralateral acoustic reflex thresholds for tonal activators using wideband energy reflectance and admittance. Journal of Speech, Language and Hearing Research 46: 128–136. 10.1044/1092-4388(2003/010) 12647893

[28] Keefe DH, Fitzpatrick D, Liu Y-W, Sanford CA, Gorga MP (2010) Wideband acoustic-reflex test in a test battery to predict middle-ear dysfunction. Hearing Research 263: 52–65. 19772907 10.1016/j.heares.2009.09.008 PMC3694582

[29] Foster JR, Haggard MP (1987) The four alternative auditory feature test (FAAF)–linguistic and psychometric properties of the material with normative data in noise. British Journal of Audiology 21: 165–174. 3620751 10.3109/03005368709076402

[30] Levitt H (1970) Transformed up-down methods in psychophysics. Journal of the Acoustical Society of America 49: 467–477. 5541744

[31] Bland JM, Altman DG (1999) Measuring agreement in method comparison studies. Statistical Methods in Medical Research 8: 135–160. 10501650 10.1177/096228029900800204

[32] Bland JM, Altman DG (2010) Statistical methods for assessing agreement between two methods of clinical measurement. International Journal of Nursing Studies 47: 931–936. 2868172

[33] Morris SB, DeShon RP (2002) Combining effect size estimates in meta-analysis with repeated measures and independent-groups designs. Psychological Methods 7: 105–125. 11928886 10.1037/1082-989x.7.1.105

[34] Garinis AC, Glattke T, Cone BK (2011) The MOC Reflex During Active Listening to Speech. Journal of Speech, Language, and Hearing Research 54: 1464–1477. 10.1044/1092-4388(2011/10-0223) 21862678

[35] De Boer J, Thornton ARD (2007) Effect of subject task on contralateral suppression of click evoked otoacoustic emissions. Hearing Research 107: 83–92. 10.1016/j.heares.2007.08.002 17910996

[36] Chandrasekaran B, Kraus N (2010) The scalp-recorded brainstem response to speech: Neural origins and plasticity. Psychophysiology 47: 236–246. 19824950 10.1111/j.1469-8986.2009.00928.x PMC3088516

[37] Brown GJ, Ferry RT, Meddis R (2010) A computer model of auditory efferent suppression: implications for the recognition of speech in noise. Journal of the Acoustical Society of America 127: 943–954. 20136217 10.1121/1.3273893

[38] Clark NR, Brown GJ, Jürgens T, Meddis R (2012) A frequency-selective feedback model of auditory efferent suppression and its implications for the recognition of speech in noise. Journal of the Acoustical Society of America 132: 1535–1541. 22978882 10.1121/1.4742745

[39] Messing DP, Delhorne L, Bruckert E, Braida LD, Ghitza O (2009) A non-linear efferent-inspired model of the auditory system; matching human confusions in stationary noise. Speech Communication 51: 668–683.

[40] Yost WA (1988) The masking-level difference and overall masker level: restating the internal noise hypothesis. Journal of the Acoustical Society of America 83: 1517–1521. 3286709 10.1121/1.395907

[41] Hartmann WM, Constan ZA (2002) Interaural level differences and the level-meter model. Journal of the Acoustical Society of America 112: 1037–1045. 12243152 10.1121/1.1500759

[42] Zhao W, Dhar S (2011) Fast and slow effects of medial olivocochlear efferent activity in humans. PLoS ONE 6: e18725. 21494578 10.1371/journal.pone.0018725 PMC3073004

[43] Cooper NP, Guinan JJ (2003) Separate mechanical processes underlie fast and slow effects of medial olivocochlear efferent activity. J Physiol 548: 307–312. 12611913 10.1113/jphysiol.2003.039081 PMC2342783

