# Supplementary figures and images for: Top-Down Influences of the Medial Olivocochlear Efferent System in Speech Perception in Noise
Source: PLoS One. 2014 Jan 20;9(1):e85756. doi: 10.1371/journal.pone.0085756 (PMC3896402; doi:10.1371/journal.pone.0085756)

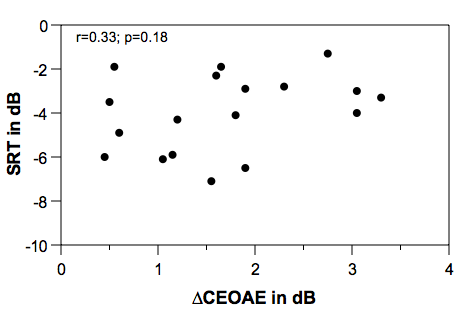

Supplement: Figure S1 — MOC inhibition in raw dB index and speech perception in noise. Bivariate scatterplot depicting the relationship between MOC reflex magnitude (ΔCEOAE) and speech recognition threshold without CAS (SRT). Pearson’s correlation coefficient (r) is inserted on top left corner of the plot. (TIFF) [file pone.0085756.s001.tiff]

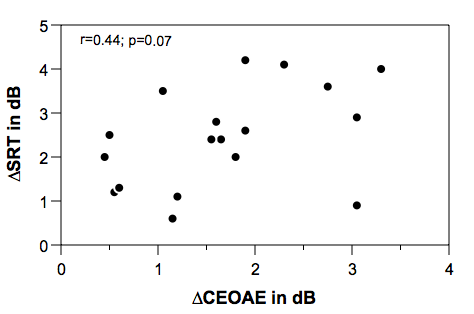

Supplement: Figure S2 — MOC inhibition in raw dB index and CAS-induced SNRSP enhancement. The CAS-induced shift in SRT is plotted as a function of MOC reflex magnitude (ΔCEOAE). Pearson’s correlation coefficient (r) is inserted on top left corner of the plot. (TIFF) [file pone.0085756.s002.tiff]
